# Lucid Dreaming Frequency Associated With Grey–White Matter Networks: An Exploratory Multimodal MRI Study

**DOI:** 10.1111/jsr.70305

**Published:** 2026-02-12

**Authors:** Nicola De Pisapia, Erdem Taskiran, Stefano Mastino, Gabriele Penazzi, Alessandro Grecucci

**Affiliations:** ^1^ Department of Psychology and Cognitive Science University of Trento Trento Italy; ^2^ Department of Education, Psychology, and Communication Sciences University of Bari Bari Italy

**Keywords:** consciousness, dream recall, lucid dreaming, mCCA + jICA, metacognition, multimodal MRI, structural brain networks

## Abstract

Lucid dreaming, defined as the experience of becoming aware of dreaming while dreaming, offers a unique window into a state of consciousness characterised by a blending of the sensory vividness of REM sleep with the self‐awareness of wakefulness. While past functional imaging has shed light on the neural activity supporting lucid dreaming, the structural brain correlates of lucid dream frequency as an individual trait varying in the normal population, remain largely unexplored. Moreover, the possibility of separating ordinary dreams from lucid dreaming has been only partially explored. In this exploratory study, we employed a data‐driven, multimodal neuroimaging approach known as mCCA + jICA, to identify joint and modality‐specific grey matter (GM) and white matter (WM) morphometric features associated with the individual differences in lucid and non‐lucid dream recall measured by a validated self‐report measure. Results revealed that lucid dreaming frequency was associated with one joint GM–WM component, encompassing frontal, temporal, parietal, and cerebellar regions implicated in metacognition, imagery, and volitional control, as well as one GM‐specific component involving visual and attentional areas including the cuneus. In contrast, ordinary dream recall frequency was associated exclusively with two WM‐specific components, showing no overlap with those linked to lucid dreaming. These findings suggest that the tendency to experience lucid dreams is rooted in distributed, structurally covarying brain systems, distinct from those underlying general dream recall. The presence of joint components supports the view that lucid dreaming depends on the integration of cortical and subcortical systems mediating self‐awareness and internal simulation.

## 1 Introduction

Lucid dreaming is the ability to become aware that one is dreaming while still immersed in the dream. Though long acknowledged in various cultural traditions and investigated sporadically in early psychology (e.g., Van Eeden 1913), lucid dreaming has only recently become the focus of systematic neuroscientific inquiry (Baird et al. 2018). Lucid dreaming is now recognised as a distinct and measurable state of consciousness, that blends the immersive sensorimotor and affective properties of REM sleep with features typical of wakefulness, metacognition, self‐reflection, and volitional control (Voss et al. 2009; Hobson 2009; De Pisapia 2021). Remarkably, lucid dreamers can even perform cognitive tasks and communicate answers to external questions without waking, demonstrating that metacognitive monitoring and working memory can operate during REM sleep (Konkoly et al. 2021).

From a phenomenological standpoint, lucid dreaming has been described as a state of consciousness in which the dreamer becomes aware of the dream while dreaming, accompanied by the possibility to control or observe its contents without waking up (Stumbrys et al. 2014; De Pisapia 2021). Empirical evidence shows that lucid dreams predominantly occur during REM sleep, yet they involve a partial reactivation of frontoparietal regions typically suppressed in this stage (Dresler et al. 2012; Voss et al. 2009). This reactivation is associated with a re‐emergence of executive functions, including attentional control, prospective memory, and reality monitoring, which are cognitive processes that, when integrated, enable the dreamer to recognise the dream state and potentially influence its development (Baird, Mota‐Rolim, and Dresler 2019; Filevich et al. 2015).

In one of the very “few MRI studies” on lucid dreaming, Baird et al. (2018) compared frequent lucid dreamers to controls, revealing significantly elevated resting‐state connectivity between the left anterior prefrontal cortex and temporoparietal association areas (including bilateral angular gyrus, middle temporal gyrus, and right inferior frontal gyrus) in frequent lucid dreamers. The heightened frontoparietal connectivity suggests that lucid dreamers have a ‘tighter’ communication between brain hubs for metacognition and self‐monitoring and regions involved in integrating multimodal information. However, in this study no significant structural differences were observed.

Lucid dreaming involves not only metacognitive awareness but also vivid perceptual experiences that occur in the absence of external sensory input, underscoring the importance of internally generated imagery. Neuroimaging studies have demonstrated that dream content, particularly visual elements, can be decoded from brain activity patterns during sleep, with significant activation observed in the visual cortex (Horikawa et al. 2013). This supports the notion that dreaming recruits visual processing areas even in the absence of retinal input. More recently, electrophysiological evidence has revealed that the onset of lucidity during REM sleep is accompanied by a surge in high‐frequency gamma activity within occipito‐temporal regions, including the precuneus, which is an area closely linked to visual imagery and self‐referential simulation (Demirel et al. 2025). Together, these findings suggest that lucid dreaming is supported by the dynamic engagement of posterior cortical networks responsible for constructing and monitoring internally generated visual content.

At a theoretical level, lucid dreaming challenges traditional dichotomies between sleep and wakefulness, suggesting that consciousness may be best conceived as a multidimensional space with fluid overlapping states rather than binary (Windt and Metzinger 2007). In this view, lucid dreaming represents a liminal state, namely a transitional, fluid configuration of consciousness where internally generated imagery is met with externally modelled cognition. This view aligns with enactive and predictive coding approaches (Friston 2010; Hobson et al. 2014), which conceptualise dreaming (and lucid dreaming in particular) as the brain's generative simulation of a virtual world governed by perceptual inferences and probabilistic self‐models. Recent theoretical work supports this view, suggesting that lucid dreaming may arise from prediction‐error signals that prompt the brain to construct a higher‐order self‐model to reconcile the dream's anomalies (Simor et al. 2022). In this sense, lucid dreaming may occur when metacognitive and predictive‐processing mechanisms register the internally generated nature of the dream experience, making it a powerful test case for theories of consciousness (De Pisapia 2024).

Despite advances in the functional characterisation of lucid dreaming, little is known about the structural neuroanatomical correlates of lucid dreaming frequency when conceived as a neurocognitive trait that varies across individuals. Some people experience lucid dreams far more often than others, yet the anatomical basis of this variability remains unclear. Although only one fMRI study (Dresler et al. 2012) has directly examined lucid dreaming, preliminary convergent evidence from resting‐state fMRI (Baird et al. 2018), EEG (Voss et al. 2009), and recent electrophysiological work (Demirel et al. 2025) likewise points to the involvement of frontoparietal circuits in this phenomenon. Some studies using univariate approaches like voxel‐based morphometry (VBM) have reported increased grey matter volume in frontopolar regions among frequent lucid dreamers, suggesting trait‐level anatomical differences (Filevich et al. 2015). However, other targeted analyses focusing on predefined regions of interest have failed to detect significant structural alterations (Baird et al. 2018). Beyond these mixed results, conventional morphometric methods remain constrained by their focus on isolated brain regions and their inability to capture the distributed network organisation of the brain or to examine how grey matter (GM) and white matter (WM) jointly support complex cognitive phenomena such as lucid dreaming.

To overcome these limitations, we employed multimodal canonical correlation analysis with joint independent component analysis (mCCA + jICA; Sui et al. 2013), an unsupervised machine learning approach that decomposes the brain into naturally grouping networks based on covarying GM and WM concentrations. Unlike traditional approaches, this method (i) captures statistical dependencies among voxels without relying on rigid, a priori parcellations, (ii) incorporates whole‐brain neural systems rather than isolated regions, and (iii) uncovers latent structural patterns without the constraints of predefined labels (Xu et al. 2009; Grecucci et al. 2025; Baggio et al. 2025). Furthermore, this network‐based decomposition approach is more coherent with contemporary network perspectives in neuroscience (Grecucci et al. 2025), allowing us to investigate how distributed structural covariance patterns across both grey and white matter modalities in relationship to individual differences in lucid dreaming frequency. Therefore, the first aim of the study is to provide evidence of joint and separate GM‐WM networks supporting lucid dreaming frequency.

Additionally, we also aim to examine ordinary dream recall frequency (DRF) to assess whether the identified components are specific to lucid dreaming or reflect more general aspects of oneiric cognition. Given the close correlation between LDF and DRF (Baird et al. 2018), yet their differing cognitive demands, a comparative multimodal analysis may help delineate the structural specificity of lucid dreaming as a distinct capacity.

In sum, in this study we adopted a data‐driven approach to identify joint GM‐WM patterns that covary with individual differences in LDF. Our goal was to uncover latent structural correlates of dreaming capacities without constraining the analysis to predefined regions of interest, thereby enabling the discovery of novel neuroarchitectural features involved in this unique phenomenon.

## 2 Methods

### 2.1 Participants

The study involved a total of 30 healthy adult participants evenly balanced by gender (15 males, 15 females), aged between 19 and 38 years. The mean age was 26.47 years (SD = 4.75) for males and 26.00 years (SD = 5.52) for females. Participants were recruited via local advertisements targeting the university community and the general population within the Trento region. Inclusion criteria for participation included being between 18 and 40 years old, native Italian‐speaking proficiency, and right‐handed. Participants were screened for neurological health, with the explicit exclusion of individuals currently undergoing psychopharmacological treatments. Exclusion criteria encompassed the presence of metal implants, metallic stitches, extensive tattoos (defined as a single tattoo exceeding 5 cm or multiple tattoos within a 20 cm proximity), and claustrophobia. To increase the likelihood of recruiting experienced lucid dreamers, additional participants were sought through dedicated online Italian lucid dreaming groups and communities. These volunteer participants underwent a preliminary screening where candidates described their lucid dreaming experiences, including frequency and level of dream control. Individuals reporting more frequent and intense lucid dreaming experiences were prioritised. Due to logistical constraints, some potential participants from distant regions of Italy could not be included in the final sample.

The experimental protocol received ethical approval from the Ethics Committee of the University of Trento. All procedures complied with the ethical standards and guidelines outlined by the Declaration of Helsinki. Written informed consent was collected from all participants prior to the initiation of the study procedures. Participants received financial reimbursement for their travel expenses to and from the neuroimaging laboratory.

### 2.2 Questionnaires

Participants completed a questionnaire assessing both their ability to recall regular dreams and their frequency of experiencing lucid dreams. The questionnaire integrated established measures from previous research (Schredl and Erlacher 2004; Baird et al. 2018). To measure DRF, we used a 15‐point Likert scale ranging from 0 (never) to 15 (more than one dream per night), following the methodology of Baird et al. (2018). Participants answered the question: ‘Approximately how often do you recall your dreams? (i.e., how often do you wake up from sleep and remember having a dream? You may—and usually do—forget them later.)’.

To assess LDF, we used the same 15‐point scale (0 = no lucid dreams to 15 = multiple lucid dreams per night), also based on Baird et al. (2018). To ensure that participants clearly understood what constituted a lucid dream, we provided them with two complementary definitions before they responded, a practice recommended in lucid dream assessment research (Schredl and Erlacher 2004; Snyder and Gackenbach 1988). The first definition stated: ‘Lucid dreaming is a special kind of dream in which you know you are dreaming while you are still in the dream. Typically, you tell yourself “I'm dreaming” or “This is a dream”’. We supplemented this with a second definition, as used in Schredl and Erlacher (2004), which emphasises control aspects: ‘During lucid dreaming, one is—while dreaming—aware of the fact that one is dreaming. It is possible to deliberately wake up, control the dream action, or passively observe the dream with this awareness’. The full questionnaire items and response options are available in the Supporting Information.

### 2.3 Data Acquisition and Pre‐Processing

Brain imaging data were acquired using a 3T Siemens Prisma scanner at the functional MRI laboratory of the University of Trento (Italy). For each participant, high‐resolution anatomical images were obtained using a T1‐weighted sequence lasting 6 min and 3 s, comprising 176 sagittal slices. The acquisition parameters included repetition time (TR) = 2530.0 ms; multiple echo times (TE): TE(1) = 1.69 ms, TE(2) = 3.55 ms, TE(3) = 5.41 ms, TE(4) = 7.27 ms; flip angle = 7°; and isotropic voxel size of 1 × 1 × 1 mm^3^. All participants underwent screening for MRI compatibility by a qualified medical professional. Eligible participants proceeded to the MRI scanning session, initially consisting of anatomical and functional imaging sequences. Anatomical data acquisition was performed during the first segment of the scanning session. Although functional imaging was also obtained, only the structural (T1‐weighted) images were utilised in this study. Quality assessments of T1‐weighted structural images were performed to identify and exclude any artefacts prior to analysis. Preprocessing was conducted using Statistical Parametric Mapping (SPM12; Penny et al. 2011) software in conjunction with the Computational Anatomy Toolbox (CAT12; Gaser et al. 2024) within MATLAB. Initially, each anatomical image was manually re‐oriented, positioning the anterior commissure at the origin. Subsequently, structural images underwent segmentation into GM, WM, and cerebrospinal fluid (CSF) using CAT12 (CAT12; http://www.neuro.uni‐jena.de/cat/). Inter‐subject registration was performed using Diffeomorphic Anatomical Registration Through Exponential Lie Algebra (DARTEL; Ashburner 2007), facilitating precise anatomical alignment across participants. After successful registration, images were normalised to the standard Montreal Neurological Institute (MNI) space, and smoothed with an 8‐mm FWHM Gaussian kernel.

### 2.4 Data Fusion Unsupervised Machine Learning

**FIGURE 1:**
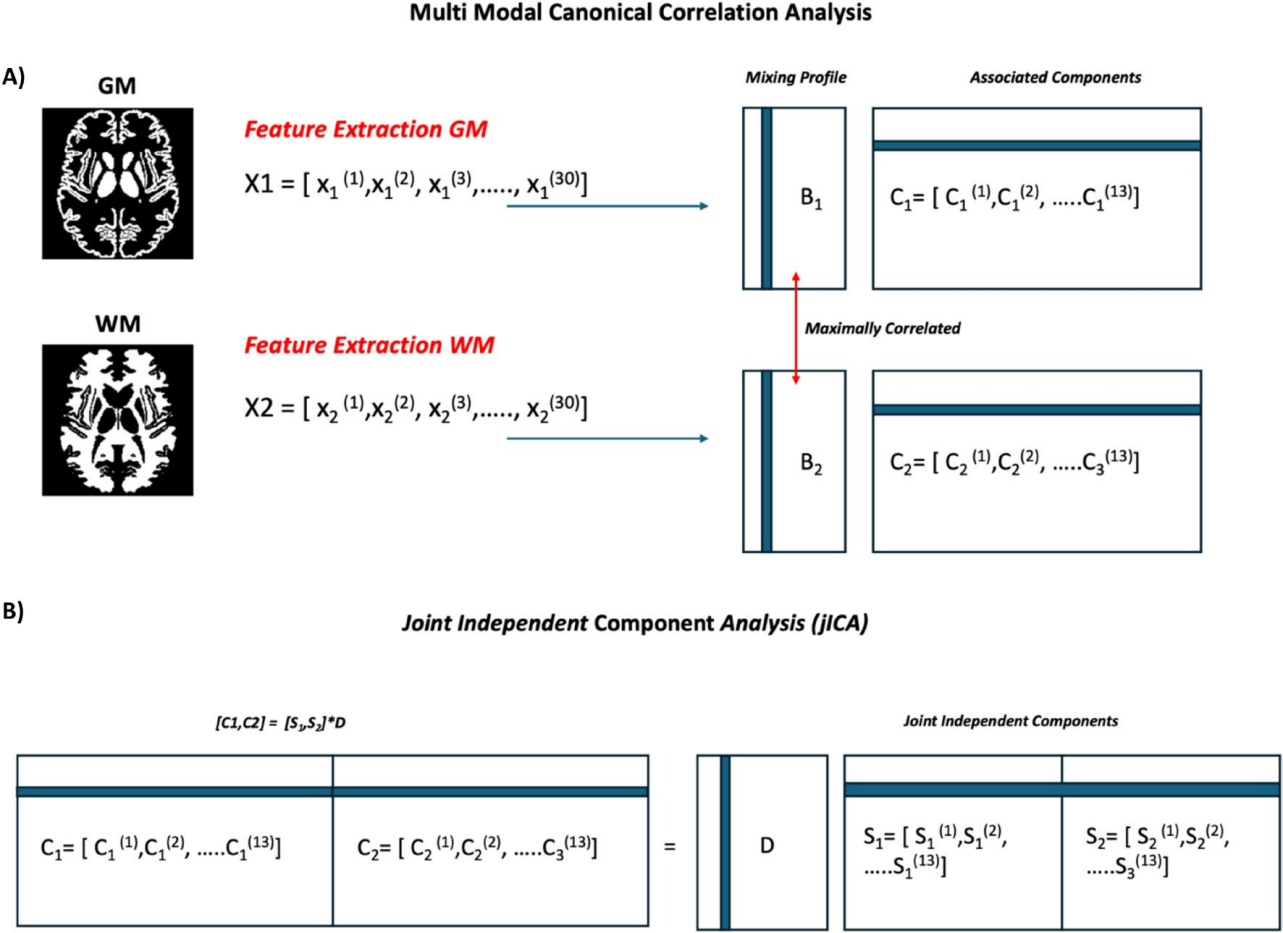
(A) Represents the first analytical step, multi‐modal canonical correlation analysis (mCCA), applied to integrate structural MRI data from Grey Matter (GM) and White. Matter (WM). Initially, features extracted from each participant's MRI scans are arranged into two modality‐specific data matrices, denoted as X_1_ (GM) and X_2_ (WM). Here, the notation indicates the number of participants involved in the study as *n* = 30, with each participant's data represented as vectors. Each vector x_1_^(i)^ or x_2_^(i)^ corresponds to a single participant's voxel‐wise GM or WM data, respectively. Mixing profiles (canonical variates matrices), represented by B_1_ for GM and B_2_ for WM, each matrix sized [30 participants × 13 components]. Associated canonical components, represented by C_1_ and C_2_, where each modality results in 13 canonical components based on the component estimation step. (B) Illustrates the second analytical stage, joint independent component analysis (jICA). Here, the canonical components matrices from each modality, C_1_ (GM) and C_2_ (WM), each containing 13 components, were horizontally concatenated into a single combined canonical component matrix [C_1_, C_2_]. [S_1_, S_2_], containing spatially independent joint sources. Each modality (GM and WM) has 13 independent spatial components. Mixing matrix (D) is a matrix sized [26 combined canonical components (13 GM + 13 WM) × 13 joint ICs], illustrating how these spatially independent sources combine to generate observed canonical components (A, B inspired by Kim et al. 2015).

To investigate structural brain correlates associated with lucid dreaming and ordinary dreaming recall frequencies, we relied on multimodal canonical correlation analysis combined with joint independent component analysis (mCCA + jICA). This unsupervised, data‐driven fusion method is particularly suited for integrating multimodal MRI data (Sui et al. 2011, 2012, 2018; Kim et al. 2015; Grecucci et al. 2024; Tang et al. 2020), as it simultaneously explores both shared and distinct covariance patterns across GM and WM modalities. Following preprocessing, GM and WM voxel‐based features were organised into separate data matrices (X_1_ for GM, X_2_ for WM; see Figure 1A), each structured as [number of participants × number of voxels] (Kim et al. 2015). The minimum description length (MDL) criterion was employed to determine the optimal number of independent components (ICs) for each dataset (Li et al. 2007). To reduce computational complexity while maintaining critical structural information, dimensionality reduction was applied to each dataset separately via singular value decomposition (SVD), retaining over 99.74% of variance for GM and 99.69% for WM (Sui et al. 2011). We then applied Multi‐Modal Canonical Correlation Analysis (mCCA) separately to the dimensionally reduced GM and WM feature matrices. This analysis decomposes each modality into two sets of matrices: canonical variates matrices (B_1_ for GM and B_2_ for WM), representing participant‐specific loading coefficients; and canonical component matrices (C_1_ for GM and C_2_ for WM), representing spatial covariance patterns across participants. The primary objective of mCCA is to identify linear combinations of GM and WM features that maximise their inter‐subject covariance. The resulting canonical correlation coefficients indicate how strongly the canonical variates from each modality correlate with one another across participants, reflecting shared structural covariance across GM and WM modalities (Sui et al. 2013; Grecucci et al. 2023; See in Figure 1A). The canonical components matrices from GM and WM modalities (C_1_ and C_2_) were concatenated into a joint matrix [C_1_, C_2_] and subsequently decomposed using joint independent component analysis (jICA) (See in Figure 1B). This step maximises the spatial independence of the joint sources, enabling identification of covariance patterns that may be either shared across both modalities or unique to each modality (Xu et al. 2009; Sui et al. 2012). To enhance the stability of the decomposition, jICA was repeated 100 times using the ICASSO algorithm, and the results were clustered and averaged. This approach validates the reliability of ICA estimations by mitigating convergence to local minima and ensuring reproducibility of the extracted components (Himberg et al. 2004; Himberg and Hyvärinen 2003; Grecucci et al. 2024). For source separation (ICA methodology), we utilised the FastICA algorithm (Hyvärinen and Oja 1997, 2000), which performs blind source separation (BSS) by maximising the non‐Gaussianity of the extracted components. Participant‐specific mixing matrices (final loading matrices; A_1_ for GM and A_2_ for WM) were computed by multiplying the canonical variate matrices by the ICA‐derived mixing coefficient matrix (B_1_ × D for GM; B_2_ × D for WM). These final loading matrices quantitatively express each participant's relative contribution to each joint independent component and were subsequently used as predictors in modality‐specific multiple regression analyses to examine relationships with LDF and ordinary dreaming recall frequency measures. The same software (Fusion ICA Toolbox: FIT) was then used to transform the components into Talairach coordinates. This is a process necessary to define the brain areas included in each component (while transforming to Talairach coordinates *z* threshold applied: *z* > 3.5). The data were then considered in their positive values and plotted in Surf Ice for visualisation (https://www.nitrc.org/projects/surce/, Rorden).

### 2.5 Statistical Analysis

Following extraction of participant‐specific mixing matrices (loading coefficients, matrices A_1_ for GM and A_2_ for WM), we conducted linear regression analyses to explore relationships between component loadings and behavioural measures of LDF and ordinary dreaming recall frequency. The participant‐specific loadings obtained from the mCCA + jICA analysis reflect individual contributions to each identified independent component (IC). For each dreaming frequency measure, we performed backward regression analyses independently for GM and WM loadings to avoid multicollinearity issues. In each backward regression model, all component loadings for a given modality were entered simultaneously as predictors, with components being iteratively removed based on significance threshold until only significant predictors remained. Components demonstrating significant associations were considered biologically meaningful.

Following established terminology (Grecucci et al. 2024; Tang et al. 2020; Sui et al. 2013; Lottman et al. 2018), we classified significant components into two categories based on their cross‐modal linked associations. Since CCA identifies maximally correlating components between GM and WM modalities across subjects (Kim et al. 2015), paired components share the same index across modalities (e.g., IC1‐GM and IC1‐WM). We defined ‘joint components’ as those component pairs where both the GM and WM components at the same index showed significant predictive capability for dreaming measures, indicating shared structural covariance patterns across modalities. Conversely, ‘modality‐unique components’ were identified when only one component of an index pair (either GM or WM) exhibited significant predictive capability while its paired component did not, suggesting modality‐specific relationships with dreaming frequencies.

## 3 Results

### 3.1 Behavioural Results

We examined the relationship between lucid dream frequency (LDF) and dream recall frequency (DRF) using Spearman's rank correlation, which revealed a significant positive association between the two measures (Spearman's ρ = 0.645, *p* < 0.001). The effect size, calculated using Fisher's *z* transformation, was 0.766, with a standard error of 0.199. Additionally, an independent samples *t*‐test was conducted to assess potential gender differences in both LDF and DRF. No significant differences between male and female participants emerged (LDF: t(28) = −0.246, *p* = 0.807; DRF: t(28) = −0.878, *p* = 0.387).

Finally, a Spearman's rank correlation analysis was conducted to examine whether age was associated with either LDF or DRF. No significant correlation between age and either LDF (*ρ* = 0.101, *p* = 0.595) or DRF (*ρ* = 0.100, *p* = 0.599).

### 3.2 Unsupervised Machine Learning Network Decomposition

**FIGURE 2:**
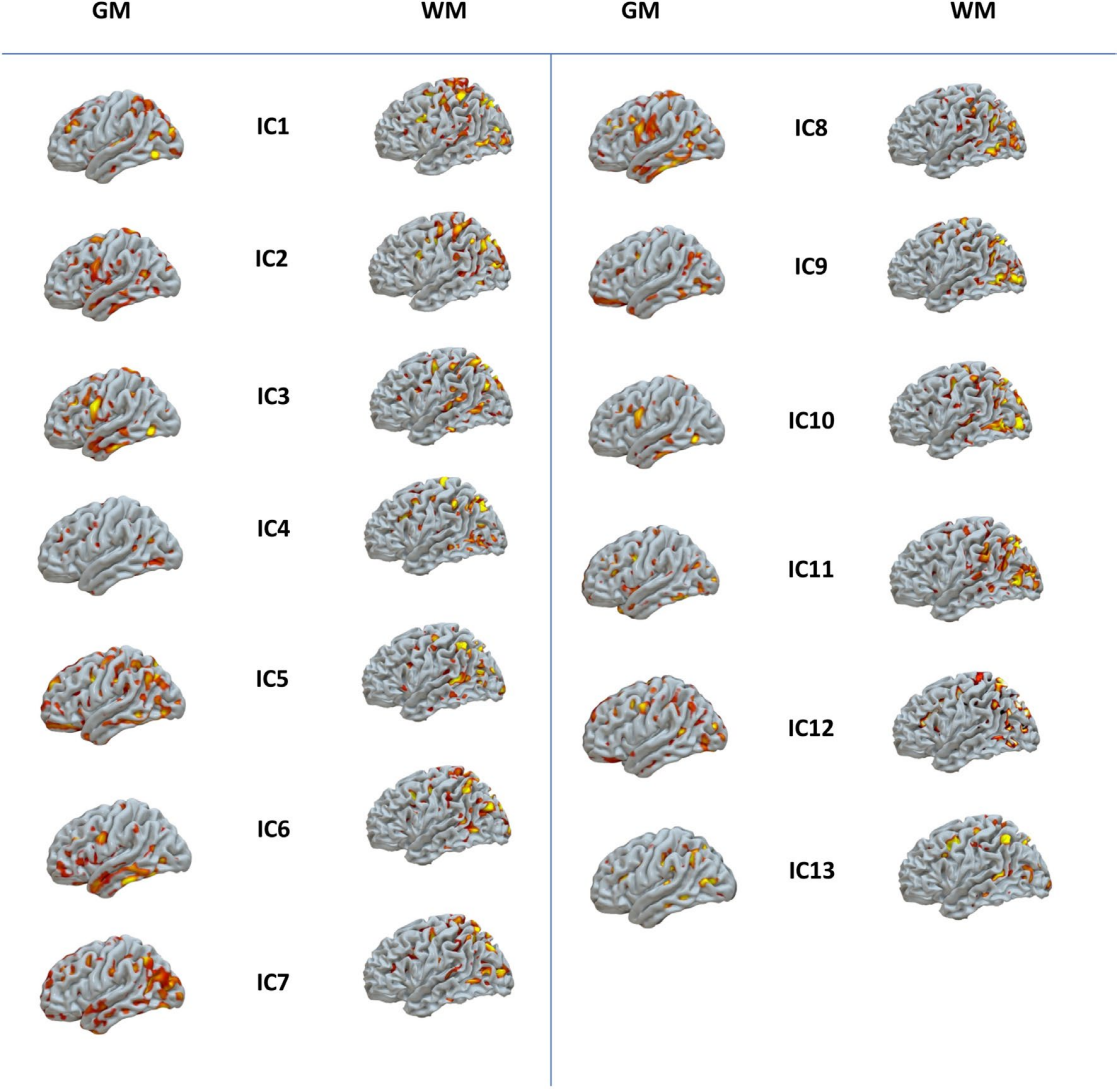
Independent covarying GM–WM networks. mCCA + jICA decomposed the brain into 13 covarying GM–WM networks. Only the positive tail of the distribution is plotted.

The MDL criterion (Li et al. 2007) was used to estimate the optimal number of independent networks, resulting in the identification of 13 independent components representing covarying GM (IC‐GM) and WM (IC‐WM) networks (Figure 2). The estimated components were visually inspected to assess data quality and exclude potential artefacts before proceeding with further analysis. No components were excluded, and all independent components (All 26 ICs; 13 for GM and 13 for WM) were retained for subsequent investigation. The regions identified in WM networks reflect WM tracts that are in close proximity to the corresponding GM regions, rather than direct functional regions themselves.

### 3.3 Brain Structural Correlates of Lucid Dreaming Frequency

**FIGURE 3:**
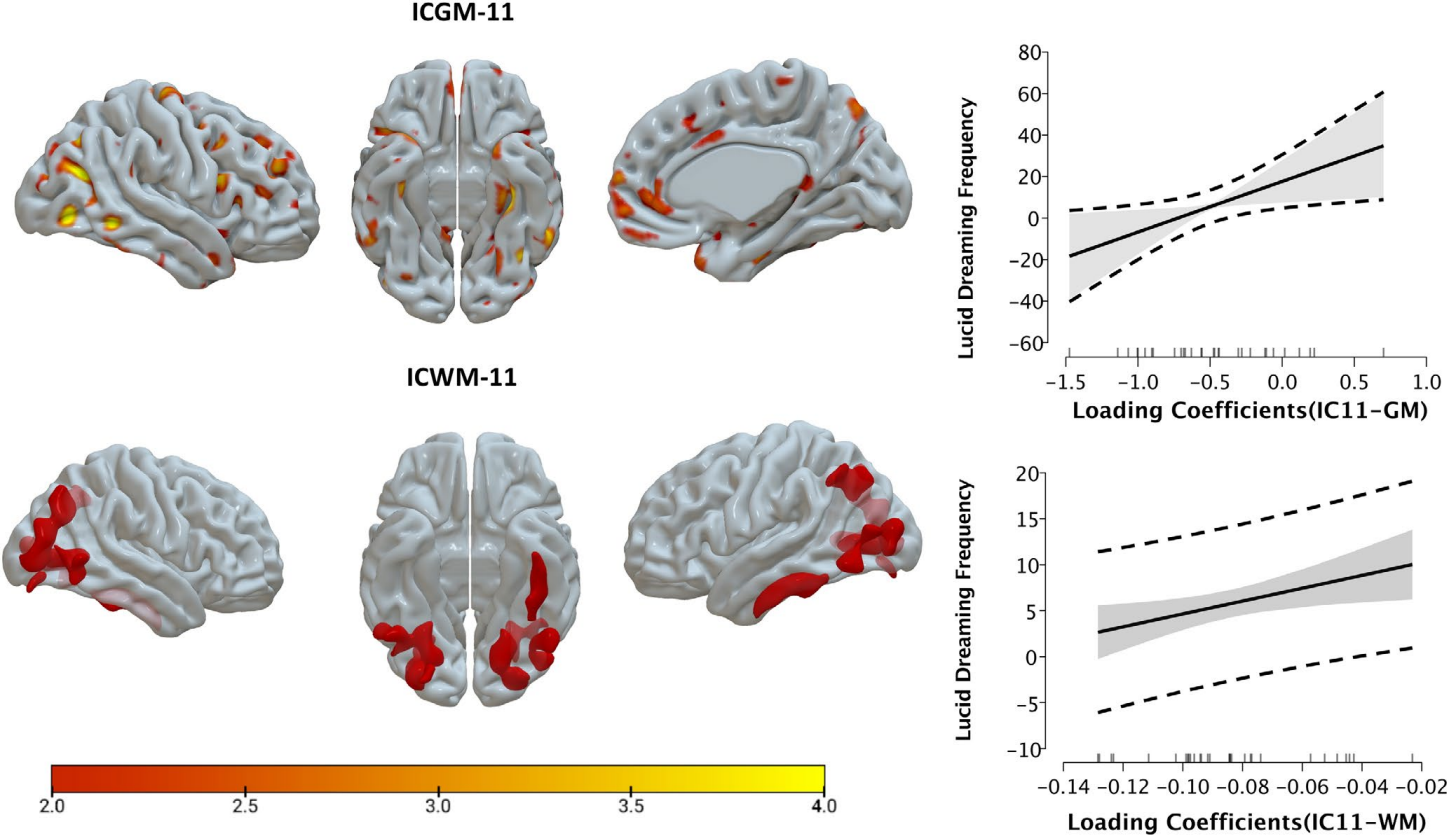
IC‐11 GM/WM Joint Components predicting lucid dreaming frequency. Top left: IC‐GM11 component maps. Warm colours indicate regions with positive loadings. Top right: Linear regression showing the positive relationship between IC‐GM11 loading coefficients and lucid dreaming frequency. Bottom left. IC‐WM11 component maps displaying in different views. Bottom right: Linear regression demonstrating the positive association between IC‐WM11 loading coefficients and lucid dreaming frequency. Spatial maps are thresholded at *z* > 2 for visualisation purposes only; this threshold is not intended to indicate statistical significance and does not reflect multiple comparison correction.

**FIGURE 4:**
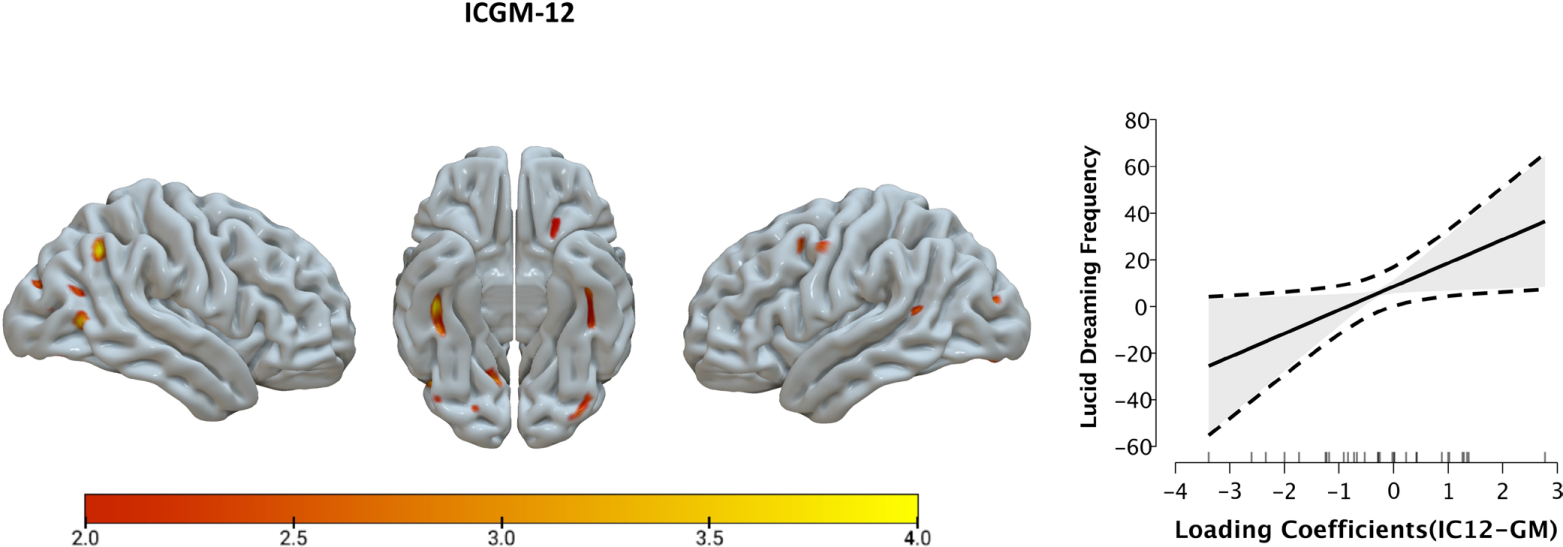
(Left) Visual representation of ICGM‐12 and its association with lucid dreaming frequency. The warmer colours in the brain plots represent positive correlational values. (Right) Plot of the regression between lucid dreaming frequency and ICGM‐12 loading coefficients. Spatial maps are thresholded at *z* > 2 for visualisation purposes only; this threshold is not intended to indicate statistical significance and does not reflect multiple comparison correction.

To examine whether structural brain components were associated with LDF, we conducted two separate backward linear regression analyses using the participant‐specific mixing matrices derived from the mCCA + jICA analysis. Age was included as a covariate; it did not significantly contribute to the models or alter the pattern of results. When considering the GM modality, the final backward regression model (*R* ^2^ = 0.31, F(2,37) = 8.76, *p* = 0.001) identified IC‐GM11 (*β* = 24.426, SE = 10.088, *t* = 2.421, *p* = 0.023) and IC‐GM12 (β = 10.060, SE = 4.474, *t* = 2.249, *p* = 0.033) as significant predictors of LDF. These results indicate that the participant‐specific mixing coefficients for both IC‐GM11 and IC‐GM12 contribute significantly to explaining inter‐individual differences in LDF. IC‐GM11 included positive results in Precentral Gyrus, Middle Frontal Gyrus, Parahippocampal Gyrus, Sub‐Angular Gyrus, Middle Temporal Gyrus, Precuneus, and Inferior Frontal Gyrus (see Figure 3 below and Table S1). IC‐GM12 showed positive results in Cuneus, Middle Frontal Gyrus, Extra‐Nuclear, and Lateral Cerebellum (see Figure 4 below and Table S2).

When considering the WM modality, the final backward regression model (*R* ^2^ = 0.24, F(1,38) = 6.36, *p* = 0.018) identified IC‐WM11 (*β* = 69.885, SE = 27.711, *t* = 2.522, *p* = 0.018) as significantly associated with LDF. IC‐WM11 included positive results in Middle Occipital Gyrus, Sub‐Angular Gyrus, Fusiform Gyrus, Precuneus, Cuneus, Superior Frontal Gyrus, Middle Temporal Gyrus, and Posterior Cerebellum.

We classified the significant components based on their cross‐modal patterns (Grecucci et al. 2024; Tang et al. 2020; Sui et al. 2013). IC11 emerged as a joint component for LDF, as both IC‐GM11 (*p* = 0.023) and IC‐WM11 (*p* = 0.018) demonstrated significant associations with LDF, representing a shared structural covariance pattern across grey and white matter that underlies individual differences in lucid dreaming frequency. In contrast, IC‐GM12 was identified as a grey matter‐specific (modality‐unique) component, as it was not correlated with any other WM component.

Supplementary analyses confirmed these associations were not confounded by demographic variables. Independent samples *t*‐tests revealed no significant gender differences in component loadings: IC‐GM11 (t(28) = 1.304, *p* = 0.203), IC‐GM12 (t(28) = −1.375, *p* = 0.180), or IC‐WM11 (t(28) = 0.295, *p* = 0.770). As noted above, including age as a covariate did not alter the significance, direction, or effect sizes of these associations.

### 3.4 Brain Structural Correlates of Ordinary Dreaming Recall Frequency

**FIGURE 5:**
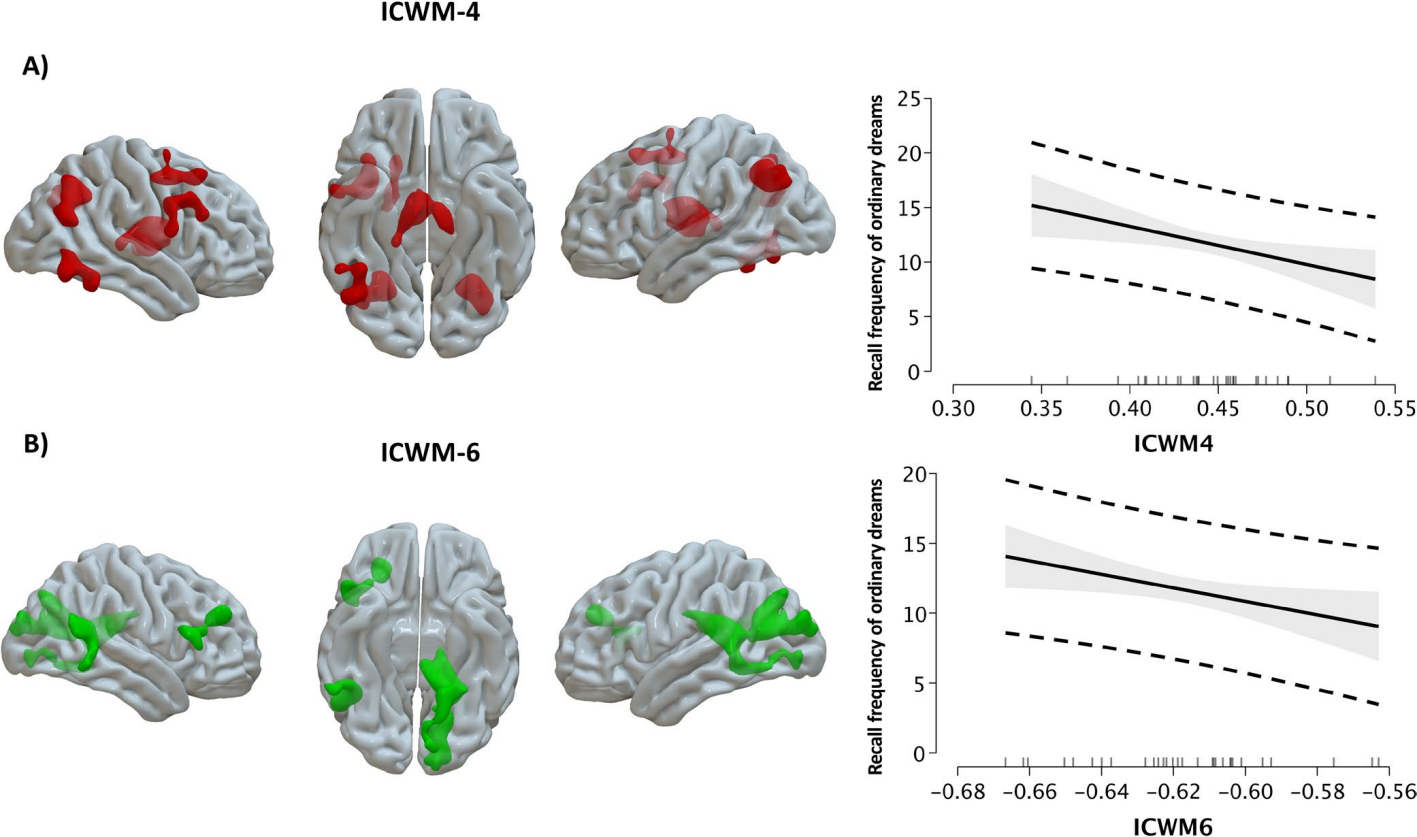
Visual representation of Ordinary Dreaming Recall Associated Modality Specific WM Components. Brain plots of ICWM4 showing left (A). The colours in the brain plots represent increased concentration. Plot of the regression between recall frequency of ordinary dreams and ICWM4 (on the top right). The solid line represents the linear relationship with a negative slope, indicating that higher ICWM4 values are associated with decreased recall frequency. Brain plots of ICWM6 showing left (B) The green colours in the brain plots represent negative correlational values. Plot of the regression between recall frequency of ordinary dreams and ICWM6 (on the bottom right). The regression shows a negative relationship with ICWM6. Spatial maps are thresholded at *z* > 2 for visualisation purposes only; this threshold is not intended to indicate statistical significance and does not reflect multiple comparison correction.

To investigate structural brain components were associated with ordinary dreaming recall frequency, we conducted similar backward regression analyses using the participant‐specific mixing matrices derived from the mCCA + jICA analysis. Age was included as a covariate; it did not significantly contribute to the models or alter the pattern of results. In contrast to the lucid dreaming analysis, the regression results revealed distinct patterns of association with ordinary dreaming recall frequency. For GM components, the backward regression analysis did not identify any significant associations. However, for WM components, the final backward regression model identified two components that were significantly associated with ordinary dreaming recall frequency: IC‐WM4 (Figure 5A) (*β* = −34.858, SE = 13.114, *t* = −2.658, *p* = 0.013) and IC‐WM6 (*β* = −48.337, SE = 20.415, *t* = −2.368, *p* = 0.025) (Figure 5B).

Since these models are negatively associated with ordinary dreaming recall frequency, the positive results in the identified brain regions indicate decreasing concentration in these areas is related to increased dream recall. IC‐WM4 showed positive results in Angular Gyrus, Precuneus, Sub‐Gyral Superior Parietal Lobule, Inferior Frontal Gyrus, Middle Frontal Gyrus, Postcentral Gyrus, Middle Temporal Gyrus, Middle Occipital Gyrus, and Thalamus. IC‐WM6 included positive results in Sub‐Gyral Middle Frontal Gyrus, Inferior Parietal Lobule, Precuneus, Lateral Ventricle, Precentral Gyrus, Middle Occipital Gyrus, and Middle Temporal Gyrus. Following the established classification criteria, IC‐WM4 and IC‐WM6 can be characterised as ‘modality‐unique components’ since they exhibited significant associations only in the WM modality and not in the corresponding index of GM.

Supplementary analyses confirmed these associations were not confounded by demographic variables. Independent samples *t*‐tests revealed no significant gender differences in component loadings: IC‐WM4 (t(28) = −0.016, *p* = 0.987) or IC‐WM6 (t(28) = 0.020, *p* = 0.984). As noted above, including age as a covariate did not alter the significance or direction of these associations.

The anatomical composition of the independent components associated with lucid and non‐lucid dream recall is detailed in Tables S1–S4 (see Supporting Information). For lucid dreaming frequency, one joint GM–WM component (IC11) and one GM‐specific component (IC12) were identified (Tables S1 and S2). In contrast, non‐lucid dream recall was associated with two WM‐specific components (IC6 and IC4) (Tables S3 and S4). Each table reports the principal grey and white matter regions, corresponding Brodmann areas, cluster volumes, and peak MNI coordinates.

## 4 Discussion

This study employed a data‐driven, multimodal neuroimaging approach to explore the structural brain networks associated with the tendency to experience lucid dreams.

By integrating GM and WM structural data using mCCA + jICA, we identified both joint and modality‐specific components that covaried with individual differences in lucid and non‐lucid dream recall frequencies. These results advance the emerging neuroscientific view of lucid dreaming as a structurally grounded manifestation reflecting interindividual differences that may underlie the capacity to be self‐conscious within the dream state.

One of the most compelling results of our analysis is the identification of a joint GM/WM component (IC‐GM11/IC‐WM11) whose expression robustly associated with LDF. This network encompassed a distributed set of regions including the middle and inferior frontal gyri, precentral cortex, precuneus, middle and superior temporal gyri, parahippocampal gyrus, fusiform cortex, and posterior cerebellum. These areas are highly consistent with the few prior functional studies highlighting the role of frontoparietal executive systems, default mode network (DMN) nodes, and visual imagery regions in lucid dreaming (Dresler et al. 2012; Voss et al. 2009; Baird et al. 2018). Notably, while Baird et al. (2018) reported no gross volumetric differences, our multimodal analysis uncovered joint structural variations in similar regions, suggesting a subtler, network‐level anatomical trait associated with lucid dreaming.

From a theoretical perspective, these DMN regions support a core set of processes central to the emergence of lucidity during dreams: metacognitive monitoring, volitional attentional control, episodic memory reactivation, and the capacity for internally generated scene construction (Christoff et al. 2016). Notably, the precuneus, a medial parietal hub involved in both DMN functioning and visuospatial simulation, emerged as a key node in both GM and WM maps. The consistent involvement across studies and imaging modalities suggests that the precuneus may act as a structural anchor for internally directed cognition, operating seamlessly across both wakefulness and sleep (Cavanna and Trimble 2006).

Interestingly, a recent high‐density EEG study found that lucid REM sleep is characterised by reduced beta power in the right TPJ and a surge of gamma in the precuneus as lucidity is attained (Demirel et al. 2025). Our structural results underline the importance of these regions, with the precuneus emerging as a key node in both GM and WM components, possibly pointing to its role as a hub for internally oriented cognition across sleep–wake states.

The WM component IC‐WM11, structurally covarying with IC‐GM11, included tracts underlying many of these same regions. This pattern suggests that the tendency to become lucid in dreams may not depend solely on localised cortical anatomy, but rather on the coherent structural integration of multimodal brain systems supporting self‐awareness, mental imagery, and volition. Such integration is consistent with predictive coding accounts of dreaming, in which the brain constructs a virtual reality from endogenous signals and evaluates its own models through simulated self‐environment interactions (Friston 2010; Hobson et al. 2014). In particular, our findings align with a very recent predictive processing account of dreaming (Simor et al. 2022). The identified joint structural network (encompassing frontoparietal executive and temporo‐occipital imagery regions) could represent the anatomical scaffold for the brain's simulation engine, generating a virtual world and simultaneously observing it. Simor et al. (2022) propose that lucid dreaming arises when neural systems support higher‐order self‐representation and predictive monitoring. The integration of cognitive‐control regions (for model generation) with sensory regions (for imagery) in our data supports this view of lucid dreaming as a self‐modelling process requiring close cooperation between frontoparietal attentional systems and posterior perceptual systems.

In this context, the hallmark higher‐order phenomenological feature of lucid dreaming, namely the recognition, from within the dream, that one is dreaming, constitutes a form of meta‐awareness that parallels mental states cultivated during meditative practices in wakefulness (Baird, Mota‐Rolim, and Dresler 2019). This metacognitive insight inherently gives rise to an ‘inner observer’ perspective and a degree of experiential detachment, traits that are also characteristic of certain meditative traditions. Indeed, the deliberate cultivation of lucidity during sleep is central to the Tibetan Buddhist practice of ‘dream yoga’ or ‘dream meditation’, which trains practitioners to sustain awareness not only during dreaming but also into dreamless sleep (Gillespie 1992; Mota‐Rolim et al. 2020). Empirically, trait mindfulness, and particularly the dimensions ‘Acting with Awareness’ and ‘Observing’ as measured by the Five Facet Mindfulness Questionnaire, have been positively associated with lucid dream frequency (Baird, Riedner, et al. 2019). In a survey study, Stumbrys et al. (2015) found that the reported frequency of lucid dreams was positively associated with higher dispositional mindfulness in wakefulness, suggesting a shared cognitive foundation. Our results revealed grey matter differences associated with lucid dream frequency in anterior and dorsolateral prefrontal regions (BA 9, 10, 46), temporo‐occipital areas (BA 36, 37, 39), and the precuneus (BA 7, 31). Notably, a systematic review and meta‐analysis of morphometric neuroimaging in meditation practitioners (Fox et al. 2014) identified overlapping structures, including the rostrolateral prefrontal cortex, inferior temporal gyrus, fusiform gyrus, and precuneus, as convergent findings of grey matter alterations observed in trained meditators. White matter changes in long‐term meditation practitioners have also been reported in the superior longitudinal fasciculus and the inferior fronto‐occipital fasciculus (Fox et al. 2014; De Filippi et al. 2022), which are also implicated in our WM modality findings for IC11.

A second, modality‐specific GM component (IC‐GM12) was also found to be associated with LDF. This component included the cuneus, middle frontal gyrus, extra‐nuclear cortex, and lateral cerebellum, which are regions implicated in low‐level visual processing, attentional orienting, and cognitive sequencing (Schmahmann 2019; Horikawa et al. 2013). The cuneus, in particular, plays a role in visual imagery and perceptual awareness, supporting the view that lucid dreaming involves an elevated capacity to monitor and assess internally generated sensory content.

The recurring involvement of cerebellar structures across both GM and WM components deserves particular attention. Once thought to be primarily motoric, the posterior cerebellum is increasingly recognised as a key node in cognitive and affective simulation networks (Schmahmann 2019). Its structural contribution here may reflect a capacity for temporal coordination, sequence prediction, or embodied presence in the dream state, all qualities frequently reported by experienced lucid dreamers (Stumbrys et al. 2014).

In contrast to the multimodal architecture associated with lucid dreaming, ordinary DRF was predicted solely by two WM‐specific components (IC‐WM4 and IC‐WM6). Both components negatively correlated with DRF and involved tracts in the precuneus, superior parietal lobule, angular gyrus, middle frontal gyrus, and thalamus, which are regions implicated in salience detection, memory access, and sensory gating (Eichenlaub et al. 2014; Vallat et al. 2018).

The lack of GM predictors, combined with the negative direction of these associations, suggests that lucid and non‐lucid dreaming are subserved by partially dissociable structural networks. Whereas lucid dreaming may reflect an anatomically integrated profile involving higher‐order metacognitive systems, ordinary dream recall could be more closely tied to connectivity patterns that gate access to dream memory traces upon awakening. This is consistent with findings that DRF can be enhanced by changes in WM integrity in medial and posterior brain regions (De Gennaro et al. 2001), but does not require the executive‐level capacities needed for lucidity.

Together, these results reinforce the conceptualization of lucid dreaming as a capacity rooted in structural neuroanatomy. While our data do not address sleep‐stage mechanisms directly, the results are consistent with recent evidence that lucid dreaming arises within REM sleep (Baird et al. 2022) and may reflect individual differences that facilitate the emergence of self‐awareness during this stage. The multimodal components identified here point to the interaction of distributed cortical and subcortical systems supporting volition, awareness, and imagery. Conceptually, this perspective can be seen as an update of early hybrid‐state formulations (Hobson 2009), situating them within a REM‐sleep framework in which lucidity emerges through the partial re‐engagement of metacognitive networks during dreaming.

Furthermore, the overlap between structural substrates of lucid dreaming and those implicated in meditation, metacognitive training, and dream induction protocols suggests that lucid dreaming may be partially plastic and trainable through practices that enhance prefrontal‐parietal integration (Fox et al. 2014; Stumbrys et al. 2012).

This possibility carries significant clinical implications, particularly for conditions such as chronic nightmares and post‐traumatic stress disorder (PTSD), which share core features of dysregulated imagery, intrusive affect, and impaired cognitive control during sleep. Both nightmares and PTSD involve a failure to modulate the emotional salience of internal experiences, often accompanied by a diminished sense of agency or perspective during dream states. Lucid dreaming, by contrast, is characterised by heightened metacognitive awareness and volitional control within the dream—a profile that directly counteracts these dysregulations. Although our study did not directly investigate training‐related neuroplasticity in lucid dreaming, the identification of stable frontoparietal and imagery‐related structural patterns in frequent lucid dreamers suggests that these networks could serve as neural targets for interventions aimed at enhancing lucidity. Strengthening such circuits through cognitive training, neurofeedback, or stimulation‐based techniques may increase the capacity to become lucid and re‐script distressing dream content, thereby offering therapeutic benefits in the treatment of nightmares and trauma‐related sleep disturbances (De Macêdo et al. 2019; Holzinger et al. 2020).

While this study offers novel and robust findings, several limitations should be noted. First, the sample size, although carefully balanced by gender and screened for neurological health, inevitably limits generalizability and statistical power. However, supplementary analyses revealed that neither age nor gender confounded our main findings: no significant gender differences emerged in any component loadings, and age showed no correlation with dreaming frequencies. Moreover, including age as a covariate in regression models did not alter the pattern of results. Second, our reliance on self‐reported dream frequencies, rather than prospective dream diaries or laboratory verification, represents a methodological limitation. However, self‐reported frequency scales are the established standard for assessing trait‐level lucid dreaming capacity in neuroimaging research (Baird et al. 2018; Filevich et al. 2015) because they capture stable individual differences over extended timeframes (months to years).

Although these limitations warrant consideration, several methodological features enhance the robustness of our findings. Unlike traditional voxel‐based morphometry, which requires correction for over 100,000 voxels, mCCA + jICA uses a dimensional reduction technique that compressed our neuroimaging data into 13 independent components while retaining > 99% of variance. This represents a substantial reduction from ~100,000 statistical tests to 13 component loadings per modality. Critically, we employed ICASSO stability analysis, repeating ICA decomposition 100 times with different random initializations; all identified components demonstrated high stability, ensuring reproducible patterns rather than random artefacts. We then applied backward stepwise regression, which tests nested models by iteratively removing non‐significant predictors rather than conducting multiple independent comparisons—this model selection procedure inherently controls for predictor multiplicity through its sequential elimination process. This overall approach aligns with established practices in multivariate fusion neuroimaging (Sui et al. 2012; Kim et al. 2015; Grecucci et al. 2024; Tang et al. 2020), where component‐level analyses with stepwise selection do not require the same multiple comparison corrections as voxel‐wise approaches. Although the regression models revealed statistically significant associations between component loadings and lucid or non‐lucid dream frequencies, these analyses were not cross‐validated. Therefore, the results should be interpreted as correlational rather than predictive, pending replication in independent samples.

Future studies employing larger and more diverse samples, objective or corroborated dream measures, and longitudinal or training‐based designs will be essential to determine whether these structural patterns reflect stable predispositions, training‐induced plasticity, or bidirectional reinforcement. Such research will not only clarify causal mechanisms but also refine the translational potential of lucid dreaming in clinical and cognitive domains.

In conclusion, this exploratory, multimodal neuroimaging study reveals that the tendency to experience lucid dreams is associated with distributed and covarying GM and WM patterns spanning executive, sensory, and metacognitive networks. By distinguishing these patterns from those related to ordinary dream recall, we provide new evidence that lucid dreaming reflects a structurally grounded form of hybrid consciousness, namely one that bridges sleep and wakefulness through integrated neurocognitive architecture. These findings open new avenues for research on the structural plasticity of conscious states and their potential applications in mental health, creativity, and the science of the self.

## Author Contributions

**Nicola De Pisapia:** conceptualisation; methodology; formal analysis; writing – original draft; supervision; project administration. **Erdem Taskiran:** formal analysis; software; data curation; writing – methods and results; visualisation. **Stefano Mastino:** conceptualization; investigation; data curation; writing – review and editing. **Gabriele Penazzi:** investigation; data curation; writing – review and editing. **Alessandro Grecucci:** supervision; methodology; software; writing – review and editing.

## Funding

The authors have nothig to report.

## Ethics Statement

This study was conducted in accordance with the Declaration of Helsinki. Ethical approval was obtained from the Ethics Committee of the University of Trento (Protocol No. 2020018). All participants provided informed consent prior to participation.

## Conflicts of Interest

The authors declare no conflicts of interest.

## Supporting information


**Data S1:** jsr70305‐sup‐0001‐supinfo.docx.

## Data Availability

The data that support the findings of this study are available on request from the corresponding author. The data are not publicly available due to privacy or ethical restrictions.
