# Supplementary material for: Lucid Dreaming Frequency Associated With Grey–White Matter Networks: An Exploratory Multimodal MRI Study
Source: J Sleep Res. 2026 Feb 12;35(4):e70305. doi: 10.1111/jsr.70305 (PMC13357955; doi:10.1111/jsr.70305)
Supplement: Supplementary file 1 — Data S1: jsr70305‐sup‐0001‐supinfo.docx. [file JSR-35-e70305-s001.docx]

**SUPPLEMENTARY INFORMATION**

**Lucid Dreaming Frequency Predicted by Integrated Gray–White Matter Networks: A Multimodal MRI Study**

**N. De Pisapia^1*^, E. Taskiran^1^, S. Mastino^1^, G. Penazzi^1^, A. Grecucci^1^.**

*^1^ Department of Psychology and Cognitive Science, University of Trento, Trento (Italy)*

**(*) Corresponding author:** [**nicola.depisapia@unitn.it**](mailto:nicola.depisapia@unitn.it)

1. **Questionnaires (EN) Original Version; Baird et al., 2018:**
2. **Ordinary Dreaming Recall Frequency:**

**Approximately how often do you recall your dreams? (i.e., how often you wake from sleep and remember having a dream. You may—and usually do—forget them later.)**

00 = Never

01 = Less than 1 dream per year

02 = 1 dream per year

03 = 2 dreams per year

04 = 3-5 dreams per year

05 = 6-8 dreams per year

06 = 9-11 dreams per year

07 = 1 dream per month

08 = 2 dreams per month

09 = 3 dreams per month

10 = 1 dream per week

11 = 2 dreams per week

12 = 3-4 dreams per week

13 = 5-6 dreams per week

14 = 1 dream per night

15 = More than 1 dream per night

1. **Lucid Dreaming Frequency:**

**Approximately how often do you recall lucid dreams (see Definition and Example)?**

**Definition of Lucid Dreaming:** Lucid dreaming is a special sort of dream in which you know that you are dreaming while still in the dream. Typically, you tell yourself "I'm dreaming!" or "This is a dream!" This realization often (but not necessarily) leads to the ability to deliberately control one’s actions or to observe passively the course of the dream with full conscious awareness, similar to the awareness you have while awake.

**Example of Lucid Dreaming:** “It was snowing gently. I was alone on the rooftop of the world, climbing K2. As I made my way upward through the steeply drifting snow, I was astonished to notice my arms were bare: I was wearing a short-sleeved shirt, hardly proper dress for climbing the second highest mountain in the world! I realized at once that the explanation was that I was dreaming! I was so delighted that I jumped off the mountain and began to fly away...”

00 = No lucid dreams yet

01 = Less than 1 LD per year

02 = 1 lucid dream per year

03 = 2 lucid dreams per year

04 = 3-5 lucid dreams per year

05 = 6-8 lucid dreams per year

06 = 9-11 lucid dreams per year07 = 1 lucid dream per month

08 = 2 lucid dreams per month

09 = 3 lucid dreams per month

10 = 1 lucid dream per week

11 = 2 lucid dreams per week

12 = 3-4 lucid dreams per week

13 = 5-6 lucid dreams per week

14 = 1 lucid dream per night

15 = More than 1 LD per night

**Questionnaires (IT) Translated Version from Baird et al., 2018:**

1. **Ricordo dei sogni**

**Approssimativamente, quanto spesso ricordi i tuoi sogni ? (vale a dire, quanto spesso ti sveglie ricordi di aver fatto un sogno. Devi contare anche quelli che ricordi appena sveglia/o ma che, normalmente, dimentichi in seguito)**

Mai

Meno di 1 sogno all'anno

1 sogno all'anno

2 sogni all'anno

3-5 sogni all'anno

6-8 sogni all'anno

9-11 sogni all'anno

1 sogno al mese

2 sogni al mese

3 sogni al mese

1 sogno a settimana

2 sogni a settimana

3-4 sogni a settimana

5-6 sogni a settimana

1 sogno a notte

Più di 1 sogno a notte

1. **Ricordo del sogno lucido**

**Denizione di sogno lucido:**

Il sogno lucido è un tipo speciale di sogno in cui sei consapevole di star sognando mentre sogni. Ingenere, si pensa "Sto sognando!" oppure "Questo è un sogno!"

. Questa realizzazione, spesso ma non necessariamente, porta alla capacità di controllare le proprie azioni in modo volontario o di osservare passivamente il corso del sogno con piena coscienza, con una consapevolezza simile a quella che si

ha da svegli.

**Esempio:**

"Nevica dolcemente. Ero solo sul tetto del mondo, scalando il K2. Mentre mi facevo strada verso l'alto, attraverso la neve che cadeva, rimasi sbalordito notando che le mie braccia erano nude: indossavo una maglietta a maniche corte, un abbigliamento poco adatto per la scalata della seconda montagna più

alta del mondo! Mi resi subito conto che la spiegazione di ciò era che stavo sognando! Ero così feliceche sono saltato giù dalla montagna e ho iniziato a volare via.."

Nessun sogno lucido

Meno di 1 sogno lucido all'anno

1 sogno lucido all'anno

2 sogni lucidi all'anno

3-5 sogni lucidi all'anno

6-8 sogni lucidi all'anno

9-11 sogni lucidi all'anno

1 sogno lucido al mese

2 sogni lucidi al mese

3 sogni lucidi al mese

1 sogno lucido a settimana

2 sogni lucidi a settimana

3-4 sogni lucidi a settimana

5-6 sogni lucidi a settimana

1 sogno lucido per notte

Più di 1 sogno lucido per notte

**Table explanations:** Coordinates were initially identified in MNI space, then transformed into Talairach space for anatomical labeling. Spatial extent is reported in cubic centimeters (cc), and the peak Z-value along with its corresponding MNI coordinates are indicated for each region. Regions not matched to the Brodmann Area atlas are marked with an asterisk (*).
